# Genetic and Epigenetic Alterations in Parathyroid Neoplasms

**DOI:** 10.30699/ijp.2022.551233.2865

**Published:** 2022-08-25

**Authors:** Seyed Amir Miratashi Yazdi, Elham Nazar

**Affiliations:** 1 *Department of General Surgery, Sina Hospital, Tehran University of Medical Sciences, Tehran, Iran*; 2 *Department of Pathology, Sina Hospital, Tehran University of Medical Sciences, Tehran, Iran*

**Keywords:** Carcinoma, Mutation, Parafibromin, Parathyroid gland

## Abstract

The exact etiology of parathyroid carcinomas (PC) is still unknown. Their associations have with several inherited syndromes or specific genetic lesions have been established. The management of PC is challenging for clinicians. The complexity of molecular phenotypes increases with tumor aggressiveness. Lack of parafibromin on immunohistochemistry staining and HRPT2 mutation would be helpful in differentiation of carcinoma from adenoma. Lack of parafibromin expression, the gene product of HRPT2 is now used as a diagnostic, prognostic, and predictive marker for parathyroid carcinoma. The epigenetic alteration, for example, DNA methylation and modifications in the chromatin structure, are known as significant events involved in the parathyroid tumorigenesis. We suggest that adjuvant genetic and epigenetic target therapy should be considered in treatment of the patients with PC.

## Introduction

The parathyroid glands are located in the back of the thyroid gland and secrete parathyroid hormone (PTH), which is needed for calcium homeostasis (1). Parathyroid glands sense the calcium level in extracellular parts via the G-protein coupled calcium-sensing receptor and secrete PTH (2). The etiology for parathyroid cancer is principally mysterious. Some familial syndromes with specific genetic mutations have been described (3). Also, patients with familial hyperparathyroidism, multiple endocrine neoplasia type 1 (MEN 1), and a history of cervical radiotherapy are at high risk for parathyroid cancer development (4). 

The incidence of benign parathyroid disease is higher in women compared to men (3-4:1 ratio) but parathyroid cancer may occur with an equivalent rate in both genders. The age at diagnosis of parathyroid cancer is earlier than benign primary hyperparathyroidism (PHPT) (5). Parathyroid cancer in young people has familial and genetic basis (6). Parathyroid carcinoma (PC) is an infrequent tumor of different behavior of aggressiveness without an existing staging system (7). 

Most parathyroid cancers release PTH and consequently induce hypercalcemia. So, parathyroid cancer's morbidity and mortality are generally due to metabolic issues (8). Increased serum PTH level is the reason for bone resorption and damage, which is acknowledged as osteitis fibrosa cystica (9). The most common manifestations are bone disease, palpable neck mass, and renal stone with increased serum calcium (10). Although diagnosis before surgery is difficult, clinicians should suspect hyperparathyroid patients with a palpable neck mass, severe hypercalcemia, a noticeable rise in serum PTH level, and metabolic complications (11).

However, in developed countries, most PHPT patients are asymptomatic (12) due to routine neck ultrasonography assessment and incidental parathyroid lesion (13). PC is a slow-growing tumor, and metastasis occurs lately in the most common sites, including lungs, cervical lymph nodes, and liver. Local recurrence in PC is usual and happens in cervical areas (14). So, PC resection with a shift of intraoperative PTH level to normal range confirmation should be considered (15). PC usually has local recurrence with adjacent lymph node involvement (16) as the decisive prognosis relates to successful complete tumor excision at the first surgery (17). Due to the variable aggressiveness of this tumor, treatment should be individualized (18). A bulky gray-white mass with local invasion is usually seen as the surgical finding of these tumors (19). 

Histomorphologic evaluation for PC consists of presence of a trabecular growth pattern; thick fibrous trabeculae; mitotic rate (>1/10 HPFs); capsular or vascular invasion; lymph node involvement; or metastasis (20). The 2022 World Health Organization (WHO) classification described the role of molecular assessment in parathyroid neoplasms (21). Also, the WHO histopathologic criteria are described for diagnosing PC now, including adjacent tissue invasion and distant metastasis (22). PC and atypical parathyroid adenoma (APA) are different in tumor biology, recurrence rate, disease-free survival, and overall survival. So, there are diverse clinical entities (23). Clinical and paraclinical findings and operative discoveries may be indicated for PC but may not be definite, especially if there is no invasion or metastasis document (24). Awareness at the time of operation is required for the malignant potential of the encountered parathyroid mass (25). So, the treatment choice for primary and recurrent PC is surgery (26). 

Severe uncontrolled hypercalcemia is the main cause of morbidity and mortality after surgery (27). Patients' gold standard of operation is en bloc resection of the tumor (28). But the worth of en bloc resection at initial surgery is still controversial because surgery discoveries cannot discriminate APA from PC definitely (29). So, radiotherapy may be useful in patients whose PC diagnosis has been proved after surgery and histopathology evaluation (30). After tumor resection, the probability of recurrence is high and may need resection metastasis several times until control hypercalcemia manifestations (31). Also, adjuvant radiotherapy may decrease the recurrence rate and is useful for local control (32). In fact, PC management is challenging for clinicians (33). The absence of a presurgery diagnosis and inappropriate surgery schedule with its influence on survival are incompletely described (34). In addition, tumor features in histopathology examination and stage prognosis of PC are significantly related to the surgeon's skill, which is correlated with preoperative diagnosis (35). Transforming a normal cell to a neoplastic cell needs numerous genetic and epigenetic mutation series (36). For example, transcription factors, including paired box-1 (PAX1), have an active role in parathyroid neoplasms (37). So, our review described genetic and epigenetic mutations related to PC for use as diagnostic markers and new therapeutic agents for these tumors (38).

Types of Parathyroid Neoplasms 

Parathyroid tumors are a common reason for PHPT, and the diagnostic workup is often straightforward in most patients (39). Serum calcium, PTH, and alkaline phosphatase (ALP) levels in patients with PC are higher than in patients with benign disease (40). Serum calcium, PTH levels, and tumor weights are meaningfully more in the PC but are not always a discriminatory method (41). PC manifestations are profound hypercalcemia and skeletal and renal problems due to hyperparathyroidism (42). However, nonfunction parathyroid cancers have aggressive behavior (43). Also, non-invasive cervical ultrasonography may suggest a diagnostic clue to PC (44). Almost entirely recognized PCs are >15 mm at the time of diagnosis (45). Parathyroid lesions with ill-defined borders and non-homogeneous echogenicity on ultrasonography are expected to be PC (46). Magnetic resonance imaging (MRI) and computerized tomography (CT) scans are specifically valuable for recognizing mediastinal and thoracic recurrences of PC (47). Established histopathological parameters for discriminating benign from malignant parathyroid masses are not presented and cannot be predicted unfavorable prognosis (48). Clinical distinction between PC and APA has critical significance in deciding the proper extent of surgery and follow up (49). 

Also, age, sex, and tumor size have uncertain effects on survival in patients with PC (50). Based on histopathology evaluation, nuclear atypia and mitotic figures are predominant in PC (51). Nevertheless, clinical follow-up of patients with marked mitotic figures on histopathology examination exhibits no evidence of recurrence or aggressive behavior. Mitotic rate is not a reliable indicator of malignant potential in parathyroid masses (52). So, these findings are not distinctive for malignancy. According to WHO criteria, PC diagnosis should be limited to tumors with adjacent soft tissues, thyroid gland, blood vessel invasion, or patients with known metastases (53). Capsular invasion is the most important histopathologic finding (54). Also, tumor nuclear DNA index is beneficial in PC diagnosis verification, but they are limited in predicting prognosis in PC (55). Patients with established metastasis have profound hypercalcemia due to high PTH levels (56). And post-operative development of PC has a high mortality (57). Some studies showed that local cervical radiotherapy may have decreased the probability of local recurrence (58). The complexity of genetic and epigenetic mutations increases tumor aggressiveness (59). Histopathology and molecular evaluation are important factors for predicting the time of recurrence (60).

## Material and Methods

Our literature review was done by an electronic search for published articles in PubMed, Web of Science, NCBI, Scopus, and Google Scholar databases. The search was done using keywords including parathyroid gland, parathyroid carcinoma, parafibromin, and mutation. We had no limitations for the date of publication. The inclusion criteria for our search consisted of those articles showing the keywords in the title or abstract. The exclusion criteria were those articles not found in English literature. A library of all used articles was made using EndNote X20.1 software. 

**Genetic Profiles**

**Fig. 1:**
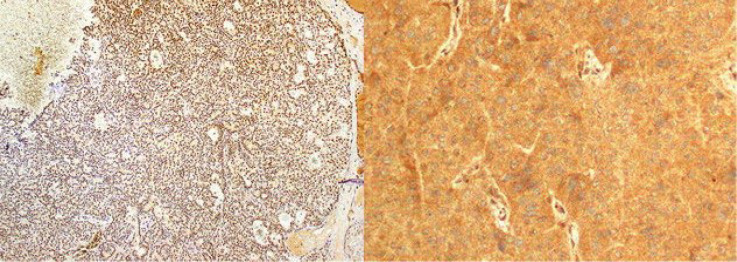
Immunohistochemical staining shows nuclear staining in the hyperplastic parathyroid gland (left) and lack of nuclear staining in parathyroid carcinoma (right) (70).

Malignant potential evaluation of parathyroid lesions in the lack of metastases can be problematic by histomorphology findings alone (61). Genome‐wide study of parathyroid tumors described some genes with transformed DNA methylation patterns as supposed to be important to benign and malignant parathyroid tumorigenesis (62). Some known genetics include deactivating and triggering alterations, and epigenetics include suppressive CpG methylation and H3K27 methylation detected in parathyroid tumors (63). The etiology of PC is unidentified, but the newly revealed HRPT2 gene (1q21-q32), a tumor suppressor gene encoding for the protein parafibromin, has been concerned in tumorigenesis (64). *HRPT2* mutation is the cause of parafibromin inactivation in the germline of patients with hyperparathyroidism-jaw tumor (HPT-JT) syndrome first time (65). Parafibromin is a helpful molecular indicator for PC (66). Parafibromin produced from *HRPT2/CDC73* gene is the human homolog of the yeast Cdc73 protein and is a section of the Protein Associated Factor1 complex (PAF1) and implicated in transcription and post-transcription control ways (67). Lack of parafibromin on immunohistochemistry (IHC) staining presents capable consequences in differentiating PC from APA and might also assist as a prognostic factor (Figure 1) (68) as well as deactivating mutations of the *CDC73* (tumor suppressor gene) have been informed in PC which is accompanying by lack of nuclear expression of parafibromin. The occurrence of the *CDC73* mutation and lack of parafibromin predict unfavorable prognosis and high recurrence or metastasis rate (69). 

IHC staining for parafibromin is difficult technically and has been arrayed by diverse researchers with variable methods and grades (71). So, parafibromin immunostaining cannot substitute genetic profile examination or be introduced as a single indicator to discrete APA from PC (72). Sporadic PCs predominantly have HRPT2 mutations that are expected to be the reason for tumorigenesis (73). Also, HRPT2 mutations indicate malignancy in both familial and sporadic parathyroid neoplasms (74). Strong immunostaining for PGP9.5 has a sensitivity of 78% for discovering PC in parathyroid tumors with some features of malignancy. PGP9.5 is positive in a parathyroid neoplasm with the HRPT2 mutation, which no expressed parafibromin (75). In HPT-JT syndrome, patients with HRPT2 mutations have a high chance for development of PC, which is supported for being responsible for HRPT2 mutations in the development of PC (76). 

Parathyroid tumors know familial MEN-1, and the gene related to MEN-1, located on chromosome 11 (11q13), may generally prevent tumor proliferation; tumors could arise from deactivating one or both of the alleles (77). The MEN1 gene produces menin, which may act in a transcription regulation pathway, including JunD and other menin-interacting proteins (78). Parathyroid neoplasms in the MEN2A syndrome are result of mutation of the RET oncogene. Also, CCND1/PRAD1 oncogene is revealed by examination of sporadic parathyroid neoplasms (79). As well as respectable studies support mutation in the CDKN1B/p27 cyclin-dependent kinase inhibitor (CDKI) gene and in other CDKI genes are cause of parathyroid neoplasms (80). Cyclin D1 protein overexpression is not restricted to neoplastic process of parathyroid gland but is also identified in the non-neoplastic process of parathyroid tissue. Cyclin D1 protein overexpression seldom exists in the normal parathyroid gland (81). Other cell cycle regulators may interfere in the parathyroid tumorigenesis, such as rearrangement and overexpression of the PRAD1/cyclin D1 gene in APA and deactivation of the retinoblastoma tumor (RB) suppressor gene in PC (82). Although the loss of expression of RB and BRCA2 genes may relate to PC, the role of 13q loss needs supplementary study as a diagnostic indicator for PC (83). 

Also, the RB gene deactivation usually happens in PC and is expected to be a significant indicator in molecular profile. Such deactivations may aid in discrimination of benign from malignant parathyroid disease and may be beneficial for diagnosis, prognosis, and therapeutic choices (84). So, RB or loss of 13q is definite for parathyroid neoplasms with aggressive behaviour (85). Gal-3 immunostaining is an important marker to support a diagnosis of PC in tumors with high proliferative index (Ki67 >6%) which involves a single parathyroid gland (86). MiRNAs are small noncoding RNAs that prevent the translation and stability of messenger RNAs (mRNAs) (87). Comparative genomic hybridization (CGH) analysis recognized chromosomal differences with recurrent loss or gain of regions in parathyroid cancers. Chromosomal imbalances have been known as a way could change the expression of miRNAs (88). Aberrant regulation of Wnt/β-catenin signaling might be significant for the growth of parathyroid tumors (89). Aberrant accretion of β-catenin is also actually common in parathyroid tumors (90). The β-catenin target gene c-myc is overexpressed in many patients with parathyroid tumors (91). β-Catenin is accompanied by PC with adjacent tissue invasion, metastasis, and poor outcome. Some new research has established that abnormal β-catenin expression, particularly in the nucleus, is an important factor in the wingless/Wnt signaling pathway (92). Recent studies proved that both gene and protein expression, including Histone 1 Family 2, amyloid β precursor protein, and E-cadherin are suitable indicators for PC and propose the occurrence of the HRPT2 mutation (93). PC has a three- to fourfold decline in p27 expression compared with adenoma. These results have advised that decreased p27 expression and high Ki-67 index may be beneficial for the difference between adenoma and carcinoma (94). 

Also, the coding region of the calcium sensing receptor gene mutations and deletions are not triggered in the tumorigenesis of sporadic parathyroid neoplasms (95). Whole exome sequencing recognized other genes consisting of mTOR, kmt2d, cdkn2c, thrap3, pik3ca, and ezh2 mutation and ccnd1 gene amplification (96). Loss of chromosome arm 1p is the most prevalent finding in molecular profile of parathyroid neoplasms recommending that 1p is the region of a tumor suppressor gene which deactivation interferes to parathyroid tumorigenesis (97). Also, the newly described tumor suppressor gene RIZ1, sited at 1p36, has appeared as a presumed indicator to be elaborated in endocrine tumorigenesis (98). So, loss of chromosome1p is meaningfully accompanied by PC and used as a discriminating marker from adenoma (99). Unlike genetic variations are involved in growth of parathyroid neoplasms including predominantly genes losses in adenoma and gains coexistent with some losses in carcinoma (100). 

In addition, some significant target proteins associated with angiogenesis and cell proliferation may be therapeutic targets in PC such as COX-1/2, Gst-π and members of sonic hedgehog pathway (101). A qualified surgeon should perform parathyroid resection in HRPT2 mutated patients and consist of visual examination of all bilateral parathyroid glands and en bloc excision of any uncharacteristic appearance glands (102). Molecular examination including specific micro-RNAs and proteins, and germ line mutations in CDC73 can detect in high risk patients but is inappropriate for presurgery evaluation due to the probable risks related with biopsy (103). In fact, the diagnostic use of molecular genetic investigates to separate benign from aggressive parathyroid neoplasms will possible need investigation of a panel of some markers or chromosomal regions, rather than any single one, to reach numerical and clinical consequences (104). A panel of IHC study including PGP9.5, galectin-3, parafibromin, and Ki67 is better than any single immunostaining and can be used as supplementary evaluation for parathyroid neoplasm diagnosis (105). Other tumor markers including P53 and BCL2 are not suitable in discriminating adenoma from carcinoma (106). 

Epigenetic Profiles

Tumor suppressor gene loss is vital in the beginning and development of cancer cells. Epigenetic alterations, for example, DNA methylation and modifications in the chromatin structure, are known as significant events that are the reason for tumor suppressor deactivation (107). DNA methylation, microRNA deregulations, and histone methylation diminishing are distinguished in parathyroid neoplasms (108). Downregulation of miRNAs is seen in PC and upregulation of miRNAs in the hyperplastic parathyroid gland. Also, miRNA profile evaluation reveals discrete miRNAs expression by tumor type, which may help as supportive indicator to discriminate benign from malignant (109). So, downregulation of miRNA is discovered in PC and is a diagnostic marker (110). However, the epigenetic status is not correlated with C19MC miRNA expression levels; decreased C19MC promoter methylation is meaningfully connected with PC and metastasis (111). 

DNA hypermethylation of CDKN2B, CDKN2A, WT1, SFRP1, SFRP2, and SFRP4 is related to decreased gene expression in equally adenoma and carcinoma (62). Loss of heterozygosity of chromosome 11 and MEN1 gene modifications in sporadic parathyroid adenoma and associates with an unrelated methyltransferase gene EZH2 has an important role in endocrine tumorigenesis (112). Hypermethylated in cancer 1 (HIC1) is a tumor suppressor gene in parathyroid glands that impaired expression of HIC1 may describe an initial occurrence through tumor progress, and only PC shows an increased methylation level and decreased HIC1 expression (113). Promoter hypermethylation in RASSSF1 and APC genes has been defined in PC, like thyroid tumors. Hypermethylated CDKN2B, P16, WT1, SFRP1, SFRP2, and SFRP4 are also seen in PC (114). Promoter hypermethylation of APC and RASSF1A are recognized in parathyroid neoplasms (115). Aberrant WNT/β-catenin signaling by loss of expression and DNA methylation of APC and accumulation of active non-phosphorylated β-catenin is detected in the examined PC (116). 

Probably, DNA methylation, histone acetylation, or deacetylation of genes identified to be linked with parathyroid tumorigenesis or unknown genes until now may reason for the unprogrammed proliferation of parathyroid cells (117). Other events, including HRPT2 intronic regions mutation, added epigenetic regulation including histone modifications, or further regulatory deactivation events, including pointing by microRNAs, may act as a reason for decreased parafibromin expression (118). Post translation histone modification was related to parathyroid tumorigenesis and the potential for new target therapy agents (119). Therefore, some studies recommend that adjuvant epigenetic therapy be noticed as an extra choice in managing patients with recurrent or metastatic PC (120).

## Conclusion

Measurement of DNA content is a suitable indicator for proper diagnosis of PC and estimates of overall survival (44). Parafibromin immunostaining could be introduced as a beneficial marker for improved PC diagnosis along with proliferation index (121). Lack of parafibromin immunostaining is nearly always linked with HRPT2 mutations, and the lack of parafibromin immunostaining powerfully suggests PC. This immunostaining could be beneficial in parathyroid neoplasms with confusing histopathological features (122). So, due to a lack of parafibromin expression, the gene product of HRPT2 is now used as a diagnostic, prognostic, and predictive marker for PC (123). No single indicative marker now distinguishes whether a parathyroid mass is a PC. Still, lack of parafibromin and other molecular changes, for example, Rb expression, and galectin‐3 over-expression, usually discriminate PC from other parathyroid neoplasms (124). Germline DNA analysis for HRPT2/CDC73 mutation is advised in all patients with parathyroid neoplasm for the possible advantage for first-degree families (125). We suggest that adjuvant genetic and epigenetic target therapy should be considered in treating PC patients. 

## Conflict of Interest

The authors declared no conflict of interest.

## Funding

None.
